# Characteristics of the Relationship of Kidney Dysfunction with Cardiovascular Disease in High Risk Patients with Diabetes

**DOI:** 10.1155/2016/7180784

**Published:** 2016-11-03

**Authors:** Attilio Losito, Loretta Pittavini, Ivano Zampi, Elena Zampi

**Affiliations:** ^1^Renal Unit, Santa Maria Della Misericordia Hospital, Perugia, Italy; ^2^Institute of Geriatrics and Gerontology, Department of Clinical and Experimental Medicine, University of Perugia, Ospedale S. Maria della Misericordia, Perugia, Italy; ^3^Department of Medicine, Hospital of Pantalla, Todi, Italy

## Abstract

We aimed at comparing the relationship of reduced estimated glomerular filtration rate (eGFR) with cardiovascular disease (CVD) and mortality between high risk patients with and without type 2 diabetes mellitus (T2DM). The cross-sectional study evaluated 16,298 participants (1,627 T2DM) acutely admitted to hospital. The longitudinal study comprised 7,508 patients (673 with diabetes and 6,835 without). eGFR was categorized into 6 stages from >90 to <15 mL/min/1.73 m^2^. Kidney dysfunction was defined by an eGFR < 60 mL/min/1.73 m^2^. Patients with T2D showed a higher prevalence of CVD (37.9% versus 23.6%; *P* < 0.001) and kidney dysfunction (25% versus 13.2%; *P* < 0.001) than in the general population. An association with CVD was found with eGFR stages from 30 to 90 mL/min/1.73 m^2^ in T2D and from <15 to 90 mL/min/1.73 m^2^ in general population, in whom the association of eGFR with coronary heart disease was in an inverse relationship (*P* < 0.01 for trend). Survival, in diabetes, was lower (*P* = 0.037) but not associated with kidney dysfunction.* Conclusions*. In a high risk population, patients admitted to hospital, the relationship of kidney function with CVD is different between T2D and the general population. Competing mortality and the presence of other major risk factors in diabetes may be responsible for this difference.

## 1. Introduction

People with type 2 diabetes mellitus (T2DM) are at high risk for cardiovascular disease (CVD) [1]. The higher prevalence of CVD in diabetes than in the general population was shown many years ago in the Framingham study and has been regularly confirmed by many studies [2, 3]. The prevalence of cardiovascular complications is influenced by several factors, some linked to demography and personal history and some linked to associated conditions [4]. In the general population the reduction in renal function is associated with a parallel increase in CVD risk, with the highest risk being found in patients with lowest renal function [5]. Also in diabetes it has been shown that the reduction of renal function represents a CVD risk factor [6]. Yet, since in people with diabetes several risk factors for CVD operate at the same time, it is not easy to single out the role played by renal dysfunction. Recently, studies have addressed the association of kidney function with the risk of different CVD in T2DM [7]. In these researches the specific role of renal dysfunction, separated from other diabetes associated risk factors, has not been investigated. Particularly, the magnitude of the association of reduced renal function with CVD has not been compared between T2DM and people without diabetes. Such a comparison might help to single out the specific role of diabetes in the complex relationship between kidney dysfunction and CVD. Patients at high risk, such as patients admitted to hospital, are a population particularly suited for an investigation on the association between kidney dysfunction and CVD, for, in these patients, CVD and renal dysfunction are highly prevalent as it is the associated mortality [8]. Most studies on the relationship between renal dysfunction and CVD in diabetes have been carried out on outpatient population and data on high risk patients are lacking. The present study was undertaken to assess the association of different degrees of reduction in renal function with CVD morbidity in T2DM comparison with a patients without diabetes. The study was carried out in people acutely admitted to hospital.

## 2. Material and Methods

### 2.1. Setting

 The study was carried out at the Santa Maria Della Misericordia Hospital in Perugia, Italy, that collects all admissions of a catchment population of approximately 200,000 and is the core clinical facility for acute CVD events in the area.

### 2.2. Study Population and Data Sources

The study is composed by two parts, cross-sectional and survival analysis. The cross-sectional study included 16,298 consecutive Caucasian people, 1,627 with type 2 DM and 14,671 without, admitted to every department of the hospital from 1 January 2007 to 31 December 2007. Patients were identified by an electronic discharge diagnosis based on International Classification of Disease (ICD) codes. People with type 2 DM were singled out by the code 250. Type 1 diabetes people were excluded. CVD examined in the association study were coronary heart disease (CHD), codes 412, 413, and 414, acute myocardial infarction (MI) code 410, ischemic stroke (IS) codes 433, 434, and 436, and hemorrhagic stroke (HS) codes 430–432. Arterial hypertension was recorded by the codes 401–405. Patients with a diagnosis of acute renal failure (ICD-9: 584) and patients treated by dialysis (ICD-9: V45.1) were excluded from the analysis. The longitudinal study included 7,508 consecutive patients (673 with diabetes and 6,835 without) of the above cohort, who lived in the Perugia area. Their survival was periodically checked by electronic searches in the registry office of the personal social security code. The follow-up ended on 31 December 2009. Mortality data were obtained from the registry office of the local health authority.

### 2.3. Measurements

The value of serum creatinine measured on admission was used for the study. A Shimadzu CL-7300 autoanalyzer was used for all determinations of serum creatinine. The analysis was performed with the automated reaction-rate method of Jaffé (BioSystems SA Costa Brava, 30 08030 Barcelona, Spain). Calibration was performed at the start and at the end of every session with a serum-based calibrator (Biochemistry Calibrator, cod. 18011, BioSystems SA). Quality control was performed with Biochemistry Control Serum Levels I and II (BioSystems SA). The performances of the measurement procedure were as follows: linearity limit: 1768 *μ*mol/L of creatinine; repeatability (within run): mean concentration 150.2 *μ*mol/L, coefficient of variation (CV) 2.9%; mean concentration 468.5 mg/dL, CV 1.3%; reproducibility (run to run): mean concentration of 150.2 *μ*mol/L, CV 3.9%; mean concentration 468 *μ*mol/L, CV 2.9%. Normal values for laboratory were as follows: men, 79.6–114.9 *μ*mol/L; women, 53.1–97.2 *μ*mol/L. Creatinine values were recalibrated to isotope dilution mass spectrometry (IDMS) [9].

For the estimation of GFR, the Chronic Kidney Disease Epidemiology Collaboration (CKD-EPI) formula was used: GFR = 141 × min(S_cr_/*κ*, 1)^*α*^ × max(S_cr_/*κ*, 1)^−1.209^ × 0.993^Age^ × 1.018 [if female] × 1.159 [if black] [10].

### 2.4. Survival

The outcome was death from any cause. Survival was assessed for people with and without diabetes together and separately.

### 2.5. Statistical Analysis

Participants were categorized according to eGFR stages (≥90 [reference group], 60–89, 45–59, 30–44, 15–29, and <15 mL/min/1.73 m^2^) following the classification recommended by National Kidney Foundation [11].

Participants' characteristics are presented as means with standard deviations or frequencies and percentage. Continuous and categorical variables were compared by ANOVA and chi-square test, respectively. Odds ratio (OR) and 95% confidence interval (CI) of the association of CVD with reduced GFR were estimated by logistic regression after adjusting for age and sex. eGFR was entered into the regression as a categorical variable. Different eGFR cutoffs were used. The first was set at 60 mL/min/1.73 m^2^. In the next analysis eGFR was entered as the above described six separated stages with ≥90 mL/min as the reference group. Finally, the analysis was repeated with the cutoff set at 90 mL/min. To calculate the trend of significant ORs across eGFR stages we considered the discrete stages as continuous variables and analyzed by linear regression. CVD was the dependent variable in the logistic regression. CVD was entered either separately for each type of disease or pooled together. Participants with and without diabetes were analyzed separately. In all analysis* P* values > 0.05 were considered nonsignificant (NS).

Survival was estimated by the Kaplan-Meier procedure. Survival curves of diabetic and nondiabetic patients were compared by log-rank statistics.

Cox proportional hazard regression was used to assess the role of the different variables on survival. Hazard ratios (HRs), adjusted for age and sex, were calculated together with their 95% CIs. In the Cox model, eGFR was entered as a categorical variable in 6 groups using the >90 mL/min as a reference group. The proportional hazard assumption was tested by Schoenfeld's residuals.

Statistical analysis was performed with STATA 11 for Windows (Stata Corp LP, 4905 Lakeway Drive College Station, TX, USA).

## 3. Results

### 3.1. Baseline Demographic and Laboratory Characteristics

General characteristics of the study population are presented categorized by the presence of diabetes in Table 1. In people with diabetes, 407 discharge diagnoses of CVD were recorded (25%). In the remaining patients, CVD diagnoses were 1,943 (13.2%). The difference was significant (chi-square 186.7, *P* < 0.001).

### 3.2. Renal Function

With respect to renal function the group with T2DM showed a significantly lower eGFR than the group without diabetes (Table 1). When the eGFR was adjusted for age, the mean was 74.6 (CI 73.4–75.9) mL/min/1.73 m^2^ in diabetes and 75.4 (CI 75.0–75.8) mL/min/1.73 m^2^ in the other patients. An eGFR < 60 mL/min/1.73 m^2^ was present in 617 (37.9%) people with T2DM and in 3,477 (23.6%) without diabetes (chi-square 168.7, *P* < 0.001).

### 3.3. Association of Pooled CVD with Kidney Dysfunction

The logistic regression, with pooled CVD as dependent variable, showed in the group without diabetes a significant association with eGFR < 60 mL/min/1.73 m^2^ (Table 2). This association was absent in diabetes. When the eGFR cutoff for reduced kidney function was set at 90 mL/min/1.73 m^2^, the regression showed an association of eGFR below that value with CVD both in people with diabetes (OR 3.505, CI 1.917–6.408, *P* < 0.001) and in those without (OR 2.173, CI 1.664–2.837, *P* < 0.001).

The analysis of the association between eGFR < 60 mL/min/1.73 m^2^ and individual CVD showed a significant association with CHD only in the group without diabetes (OR 1.361, CI 1.171–1.581, *P* < 0.001). In the analysis with eGFR entered as 6 separate stages, different results between diabetes and nondiabetes were found (Table 3). In diabetes, a reduction of eGFR below 30 mL/min/1.73 m^2^ was not associated with CVD, while in the group without diabetes a significant association was present across all stages of kidney function reduction.

### 3.4. Association of Individual CVD with Kidney Dysfunction

**Figure 1:**
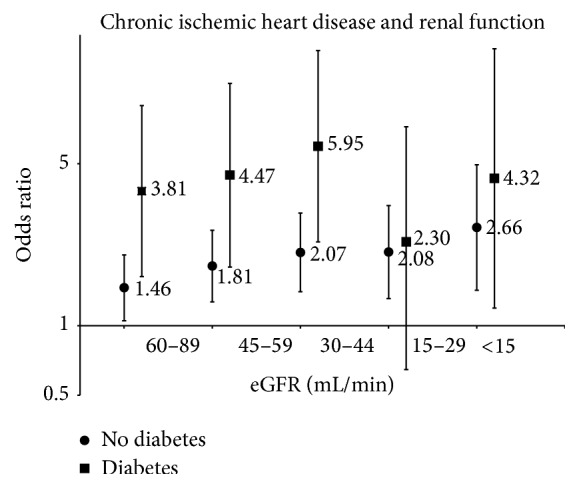
Association reduced of eGFR with CVD in patients with diabetes and patients without. The ORs for 5 different stages of eGFR are shown with their 95% CI. In patients without diabetes there is a significant linear increase in ORs for chronic ischemic heart disease as eGFR declines to <15 mL/min/1.73 m^2^ (*P* < 0.001). In patients with diabetes the trend is not statistically significant.

The logistic regression was also performed for the individual CVD with large enough numbers to allow a meaningful analysis. This analysis showed that, in patients with CHD, the relationship with reduced eGFR was different between people with diabetes and those without, in whom a significant trend of increase in ORs from higher to lower eGFR was observed (*r* ^2^ = 0.92, *P* = 0.009) (Figure 1). This trend was not observed in T2DM. IS was associated only with eGFR 60–89 mL/min/1.73 m^2^ in people with diabetes, while, in those without diabetes, the association was present with eGFR from 89 through 30 mL/min/1.73 m^2^ (Table 4). Hemorrhagic stroke was not associated with reduced kidney function in either group.

### 3.5. Survival Analysis

During the follow-up (26.7 ± 8.9 months), 1,148 deaths were recorded, 120 in the group with diabetes and 1,028 in the group without diabetes. Median survival was 31.2 and 32.9 months in people with and without type 2 DM, respectively (log-rank 4.368, *P* = 0.037).

The Cox analysis, in absence of diabetes, produced a predictive model for mortality including age, sex, and eGFR (Table 5). In this model, patients with an eGFR 15–29 mL/min/1.73 m^2^ showed a 43% higher risk of mortality than those with an eGFR > 90 mL/min/1.73 m^2^. In presence of diabetes, the reduction of eGFR of any degree was not significantly associated with mortality.

## 4. Discussion

In our study, people with diabetes show a higher prevalence of reduced renal function and CVD than patients without diabetes. Diabetic patients in our study represent a high risk group. This is shown not only by the significant shorter survival than those without diabetes, but also for their causes for hospitalization. In fact during the 12 months of observation MI was detected in 1.5% of diabetic patients, twice the value of 0.82% per year found in recent epidemiological studies in [12]. The same goes for stroke, present in 6.9% in our cohort, a much higher finding compared to what is generally found in average risk diabetic patients [13].

We have found that diabetic patients present a different relationship of CVD with reduced renal function. In fact, using eGFR < 60 mL min^−1^ 1.73 m^−2^ as a cutoff, a commonly used index for reduced kidney function [14], in diabetes we did not find the association between kidney dysfunction and CVD shown in individuals without diabetes. Furthermore, in the analysis for individual CVD in patients without diabetes, ORs of the association with CHD increase progressively with the reduction of eGFR. In T2DM this trend was absent.

There were differences also in the survival analysis. This showed, in absence of diabetes, an association between certain degrees of reduction of kidney function and mortality. In diabetes the mortality was higher than in the group without T2 DM. Furthermore, at variance with this group, in diabetes no relationship of mortality with reduced eGFR was found.

These findings are new and broaden our knowledge on the association between kidney function and CVD complications in diabetes. Our results suggest that the risk factors present only in diabetic patients play a more important role than kidney dysfunction in the association. with CVD. Yet it must be underlined that the patients we studied represent a high risk group, as shown by the high mortality rate. This high risk state may explain the higher prevalence of reduced kidney function we have found, compared with that of previous Italian studies, dealing mostly with outpatients populations. While in US and UK, among adults with a diagnosis of diabetes, CKD prevalence was ~40% [14, 15], in Italy, a survey on a population of patients attending diabetes clinics reported a prevalence of ~19% [16]. We found that ~38% of people with T2DM and 21.3% of patients without diabetes, admitted to hospital, had an eGFR < 60 mL/min/1.73 m^2^.

Also the prevalence of CVD in diabetes was much higher than in the group without diabetes: 25% versus 13.2%. This finding is close to the 23.2% reported in the recent Italian RIACE study, which was not limited to high risk diabetic patients [17]. In our study, to assess kidney function, we used only eGFR. This represents a limitation. In fact, in most of the above studies, eGFR was associated with albuminuria measurement. This increases the probability of detecting a renal dysfunction. The lack of data on albuminuria in our study may explain, at least in part, the difference in results with previous reports.

In our cohort, in T2DM, also the relationship between reduced kidney function and CVD was different from the group without diabetes. The results obtained with pooled CVD were confirmed in the analysis of individual CVD: IS and CHD. Particularly interesting are the findings in CHD, where the significant increase in the ORs of the association with different stages of reduction in eGFR was not observed in diabetes. Furthermore, in T2D, the association with reduced eGFR was much stronger in CHD than in IS. In the general population the association of kidney dysfunction with IS is an inconstant finding [18, 19]. In the present study in T2DM, eGFR < 60 mL/min was not associated with IS. In diabetes, a weaker relation of reduced eGFR to cerebrovascular disease than in the general population was shown previously in an Italian study [16]. This different relationship with kidney dysfunction between CHD and IS may be explained by the difference in involved vascular beds. In fact a maladaptive arterial remodeling has been suggested, consequence of kidney dysfunction, acting in coronary arteries and not at carotid artery level [18]. Previous reports have proposed that nonalbuminuric renal impairment, as a manifestation of prevailing renal macrovascular involvement, would be more frequently associated with coronary atherosclerosis or all-cause mortality [20, 21].

People with diabetes, in our cohort, represent a high risk sample. Therefore the difference in CVD association with the rest of population may be also explained by a competing mortality that, preceding the study, might have selected examined patients. The higher mortality, we found in diabetes, is in keeping with this hypothesis.

On the whole, our study suggests that, in a high risk population, the role of kidney dysfunction as a risk factor for CVD is different between diabetic and nondiabetic patients. The risk factors for CVD present only in diabetes may be responsible for this difference [22, 23]. Their presence may act either synergically or competitively with kidney function, altering its association with CVD.

### 4.1. Limitations

Although our results are strengthened by centralized laboratory measurements and homogeneous diagnostic criteria they are applicable only to high risk patients and not to the ordinary outpatients with diabetes. There is a large difference in the number of patients with and without diabetes, although it reflects the actual proportion of admitted patients. Age and sex differences between the two groups were taken into account performing the analysis after adjustment for these variables.

Furthermore there are limitations in the measurements and in the risk factors analyzed. The single value of serum creatinine is a limitation of our study since it may introduce bias in the assessment of the association of CVD with reduced kidney dysfunction [7]. Among cardiovascular risk factors, we analyzed only hypertension as a discrete variable. The lack of blood pressure values and pharmacological treatment represent another limitation of our study.

## 5. Conclusions

In summary, our study shows that in high risk patients with diabetes the reduction of eGFR is highly prevalent but the relationship of kidney dysfunction with CVD is different from that observed in people without diabetes.

## Figures and Tables

**Table 1:** Characteristics of studied patients.

	Diabetes	No diabetes	*P*
Number	1,627	14,671	
Sex (men%)	58.20%	50.3	<0.001
Age (years)	65.0 ± 19.8	57.0 ± 22.7	<0.001
Hypertension	509 (31.2%)	2037 (13.9%)	<0.001
MI	25 (1.5%)	102 (0.7%)	<0.001
CHD	211 (12.9%)	920 (6.2%)	<0.001
Angina	4 (0.2%)	40 (0.3%)	N.S.
TIA	27 (1.6%)	144 (0.9%)	0.009
IS	113 (6.9%)	536 (3.6%)	<0.001
HE	27 (1.6%)	201 (1.3%)	N.S.
S. creatinine (*μ*mol/L)	111.4 ± 69.8	97.2 ± 53.0	<0.001
eGFR (mL min/1.73 m^2^)	67.8 ± 30.1	76.1 ± 34.3	<0.001
eGFR 60 < mL min/1.73 m^2^	617 (37.9%)	3477 (21.3%)	<0.001
*eGFR stages *	6	6	<0.001
>90 mL/min/1.73 m^2^	255 (15.6%)	2956 (20.1%)	0.014
60–89 mL/min/1.73 m^2^	734 (45.1%)	8238 (56.1%)	<0.001
45–59 mL/min/1.73 m^2^	346 (21.2%)	3477 (23.7)	<0.001
30–44 mL/min/1.73 m^2^	170 (10.4%)	2202 (15.0%)	<0.001
15–29 mL/min/1.73 m^2^	70 (4.3%)	831 (5.7%)	<0.01
<15 mL/min/1.73 m^2^	31 (1.9%)	124 (0.8%)	<0.001

MI: myocardial infarction; CIHD: chronic ischemic heart disease; TIA: transient ischemic attack; IS: ischemic stroke; HE: hemorrhagic stroke.

**Table 2:** Association of pooled CV disease with eGFR < 60 mL/min.

	Diabetes, CV number: 407			No diabetes, CV number: 1,943		
	OR	95% CI	*P*	OR	95% CI	*P*
Age	1.033	1.024–1.042	<0.001	1.045	1.041–1.049	<0.001
Sex (M)	1.839	1.414–2.393	<0.001	1.976	1.762–2.217	<0.001
Hypertension	0.778	0.601–1.007	NS	1.654	1.458–1.876	<0.001
eGFR < 60 mL min^−1^ 1.73 m^−2^	0.872	0.675–1.126	NS	1.29	1.141–1.459	0.013

**Table 3:** Association of grouped CV disease with stages of eGFR.

eGFR stages	Diabetes (407)				No diabetes (1,943)			
	*n*	OR	95% CI	*P*	*n*	OR	95% CI	*P*
≥90 mL/min/1.73 m^2^	18	—	—	—	94	—	—	—
60–89 mL/min/1.73 m^2^	211	3,379	1.841–6.201	<0.001	1024	1,908	1.471–2.475	<0.001
45–59 mL/min/1.73 m^2^	105	3,105	1.604–6.014	<0.001	512	2,203	1.659–2.921	<0.001
30–44 mL/min/1.73 m^2^	57	3,316	1.633–6.737	<0.001	213	2,027	1.475–2.781	<0.001
15–29 mL/min/1.73 m^2^	10	1,226	0.481–3.123	NS	75	1,850	1.251–2.733	0.002
<15 mL/min/1.73 m^2^	6	1,595	0.509–4.991	NS	25	2,116	1.219–3.672	0.008

^*∗*^Obtained by logistic regression with eGFR ≥ 90 mL/min/1.73 m^2^ as reference group.

**Table 4:** Association between stages of eGFR and ischemic stroke.

eGFR stages	Diabetes, IS number: 113			No diabetes, IS number: 536		
	OR	95% CI	*P*	OR	95% CI	*P*
60–89 mL/min/1.73 m^2^	3.539	1.192–10.562	0.023	3.209	1.883–5.465	<0.001
45–59 mL/min/1.73 m^2^	2.831	0.876–9.154	NS	3.44	1.956–6.052	<0.001
30–44 mL/min/1.73 m^2^	2.873	0.825–1.001	NS	2.369	1.267–4.431	0.007
15–29 mL/min/1.73 m^2^	0.96	0.160–5.762	NS	0.947	0.373–2.355	NS
<15 mL/min/1.73 m^2^	°			1.860	0.599–5.771	NS

°Numbers too small for the analysis.

**Table 5:** HRs of AC mortality in different stages of reduction of eGFR^*∗*^.

eGFR (mL/min/1.73 m^2^)	Diabetes, patients number: 673			No diabetes, patients number: 6,835		
	HR	95% CI	*P*	HR		*P*
60–89	0.487	0.231–1.0281	NS	0.433	0.341–0.550	<0.001
45–59	0.580	0.266–1.265	NS	0.471	0.361–0.614	<0.001
30–44	1.157	0.524–2.552	NS	0.759	0.569–1.010	NS
15–29	0.849	0.318–2.269	NS	1.430	1.036–1.973	0.029
<15	2.665	0.965–7.360	NS	1.236	0.699–2.185	NS
Age	1.074	1.051–1.099	<0.001	1.071	1.065–1.077	<0.001
Sex (male)	0.810	0.551–1.190	NS	1.386	1.221–1.573	<0.001

^*∗*^Obtained from Cox regression analysis.
